# When Taekwondo Referees See Red, but It Is an Electronic System That Gives the Points

**DOI:** 10.3389/fpsyg.2021.787000

**Published:** 2021-12-13

**Authors:** Gennaro Apollaro, Coral Falcó

**Affiliations:** ^1^School of Sport Sciences and Exercise, Faculty of Medicine and Surgery, University of Rome “Tor Vergata”, Rome, Italy; ^2^Department of Sport, Food and Natural Sciences, Western Norway University of Applied Sciences, Bergen, Norway

**Keywords:** color, red, electronic protector, technology, fair play, taekwondo

## Abstract

Previous studies in taekwondo have considered the use of the manual scoring system or the electronic system with only the use of the electronic body protector. The objective of this study was to analyze the relationship between the color protectors and success in 1,327 taekwondo matches from six World Grand Prix Series of two 4-year Olympic periods when electronic body and head protectors are used. In the total sample, the results did not show a relationship between the match outcome and the color of the protectors (*p* = 0.97, *C* = 0.001). For the individual six editions, the results showed a positive and strong relationship between wearing blue protectors and winning matches and one between wearing red protectors and winning matches (*p* = 0.001, *C* = 0.19; *p* = 0.001; *C* = 0.19). Regarding the weight categories, 8 and 5 of 48 showed higher percentages of blue and red winners, respectively. Regarding sex, male competitors showed a positive relationship between blue color and winning the match in 6 of 24 weight categories, and wearing red and winning the match was shown in 2 of 24 weight categories. Female competitors showed a positive relationship between blue color and winning the match in 2 of 24 weight categories, and wearing red and winning the match was shown in 3 of 24 weight categories. When it comes to the influence of being a seeded athlete, the results did show a significant confounding effect on the color of the protectors worn by the winner of the match in 2 of 13 weight categories in which a color effect was observed (*p* = 0.02, *C* = 0.28; *p* = 0.02, *C* = 0.28). In conclusion, wearing red does not provide a higher chance of winning the match. It seems that seeing red has a stronger effect than wearing red, especially in male contenders. Moreover, being a seeded athlete does not explain the result of the match. It seems that the introduction of the electronic helmet protector, in addition to the electronic body protector, made the scoring system more objective, decreasing the advantage of wearing red in winning matches.

## Introduction

Since Hill and Barton (2005) published an article outlining that those wearing red have an advantage in combat sports, research on the color effect in sports has experienced a tremendous increase (Goldschmied et al., 2020). The majority of studies have focused on the colors red and blue (Mehta and Zhu, 2009) because they are two of the three primary colors and, in humans, have been related to psychological arousal (where red is stimulating and exciting, whereas blue is calming and relaxing) or physiological strength (where red increases physical force, whereas blue has a minimal effect). In adults, it seems that blue is a more preferred color than red, while red draws attention more strongly. Some studies have suggested that red enhances task performances compared with blue (Elliot and Aarts, 2011); others have found exactly the opposite results (Elliot et al., 2007). Red is commonly associated with danger and mistakes (e.g., incorrect answers are marked by red ink) or with avoidance motivation (due to its link with dangers and mistakes, which makes individuals pay more attention to avoid making mistakes and falling into danger). By contrast, blue is commonly associated with openness and peace (e.g., the sky and the sea both are blue; Elliot et al., 2007), which induces an approach of motivation (signals a benign environment that encourages individuals to use innovative strategies in a more explorative manner). Thus, red and blue colors can induce alternative motivations through different associations related to them (Mehta and Zhu, 2009). Red should enhance performance on detail-oriented tasks (e.g., recall tasks that require focused, careful attention) while blue should enhance performance on creative tasks (e.g., word associations, IQ tests, and tests of analogical reasoning, which assess divergent thinking and memory resources).

From the color-in-context theory (which draws on social learning theory and the field of biology), color has multiple associations and effects that differ as a function of context (Elliot and Maier, 2012). Context can include domain (e.g., red has positive associations in the attraction domain but negative associations in the achievement domain such as cognitive task performance) and type of task (e.g., red has positive effects on low-level, detail-oriented tasks but negative effects on high-level tasks that require mental manipulation; Elliot and Maier, 2014). At present, research has focused on investigating the presence of color influences in sports such as basketball (Goldschmied and Spitznagel, 2021), boxing (Gülle et al., 2016), cycling (Mentzel et al., 2021), ice hockey (Webster et al., 2012), judo (Dijkstra et al., 2018), jumping (Lam et al., 2017), rugby (Piatti et al., 2012), running (Mentzel et al., 2019), football (Attrill et al., 2008), taekwondo (Hill and Barton, 2005; Hagemann et al., 2008; Carazo-Vargas and Moncada-Jiménez, 2014; Falcó et al., 2016), wrestling (Hill and Barton, 2005), or Wushu Sanda (Vasconcelos and Del Vecchio, 2017). In this sense, there is evidence of a beneficial color effect, most for red color, in colored uniforms on sporting outcomes (Hill and Barton, 2005; Attrill et al., 2008; Piatti et al., 2012; Dreiskaemper et al., 2013; Sorokowski et al., 2014; Krenn, 2015; Vasconcelos and Del Vecchio, 2017), while other researchers did not find a benefit wearing a special color or even contradictory findings (Furley et al., 2012; Allen and Jones, 2014; Carazo-Vargas and Moncada-Jiménez, 2014; Goldschmied and Spitznagel, 2021).

Closer inspection of the methodology used in these studies (Goldschmied and Lucena, 2018), shows that various of those studies did not differentiate between wearing red [Feltmann and Elliot, 2011; Ten Velden et al., 2012; Dreiskaemper et al., 2013; see also Furley et al. (2012) and Lam et al. (2017)], perceiving a colored environment (Payen et al., 2011), viewing red on an opponent (Krenn, 2014), or some combination of all these factors. In this sense, examining the combination of wearing and perceiving colored equipment, Feltmann and Elliot (2011) found that participants imagining themselves wearing red in a taekwondo match had enhanced self-perception of their own dominance and threat, whereas perceiving an opponent in red enhanced the perception of their dominance and threat.

Discussion about wearing and perceiving effects of colors has been biased by not taking into account the impact on the judgments or decision-making of referees (Hagemann et al., 2008; Carazo-Vargas and Moncada-Jiménez, 2014; Krenn, 2014; Sorokowski et al., 2014). Those studies found that referees systematically favored or gave more points to the athlete competing in red than the one competing in blue. Therefore, in an effort to increase the fairness of the scoring process in taekwondo, the World Taekwondo (WT) and the International Olympic Committee (IOC) introduced electronic body protectors in the Olympic competition in London 2012 (International Olympic Committee, 2012). Falcó et al. (2016) analyzed the relationship between the color protector and success in taekwondo matches in the Qualification Championships and in the London 2012 Olympic Games when electronic body protectors were used. The results showed a significant relationship between wearing a red electronic protector and winning the match in the Asian and the European Qualification Tournaments. For gender and weight categories, there was no clear color effect, but a significant association was found between wearing red and winning the match in the female featherweight category (both Olympics and qualifiers). The authors highlighted the need to continue with the implementation of a fully objective system that includes the electronic registration of points for all the areas in which it is permitted to score in taekwondo, such as the electronic helmet. This additional electronic device was first used in a world-class competition at the Manchester 2014 World Grand Prix Series (WGPS) 3 (World Taekwondo, 2014a), systematically in subsequent editions and in the Rio 2016 (International Olympic Committee, 2016) and Tokyo 2020 (International Olympic Committee, 2020) Olympic competitions.

In line with the above, the objective of this study is to analyze the relationship between the color protectors and success in taekwondo matches from six WGPS of two 4-year Olympic periods when electronic body and head protectors are used. Due to the further introduction of the electronic helmets, the number of wins in these competitions by athletes in blue should be, throughout an entire edition, similar to (or the same as) the number of wins scored by athletes in red uniforms. In addition, it will also be analyzed the confounding effect of a competitor being a seeded athlete; the hypothesis is that the fact of being the seeded athletes, if a color effect should occur, would not explain a higher percentage of victories of one color than the other, as the WT has introduced the qualification system for the Olympic Games through world ranking after the London 2012 Olympics (World Taekwondo, 2015) and the WGPS are the closed-numbered event introduced in 2013 in which the top 31 athletes of the world ranking and 1 athlete from the host country of the edition per weight category take part (World Taekwondo, 2014b).

## Materials and Methods

### Participants

This study included a total of 1,327 matches, corresponding to 6 WGPS: the WGPS-1 (held in Moscow, August 14–16, 2015; *N* = 235), the WGPS-2 (held in Samsun, September 18–20, 2015; *N* = 213), the WGPS-3 (held in Manchester, October 16–18, 2015; *N* = 207), the WGPS-1 (held in Rome, June 1–3, 2018; *N* = 231), the WGPS-2 (held in Moscow, August 10–12, 2018; *N* = 211), and the WGPS-4 (held in Manchester, October 19–21, 2018; *N* = 230). Data were collected from publicly available online sources (http://m.worldtaekwondo.org/competition/list.html?mcd=A01&sc=re), ensuring the anonymity and privacy of individual athletes. The use of data from open-access sites has been previously described in other studies (Franchini and Julio, 2015; Franchini et al., 2020), and there are no ethical issues involved in the analysis and interpretation of the data used as these were obtained in a secondary form and not from direct experimentation.

### Measures

In the analysis, the color (blue or red) of the protectors (Dae do®, Spain: 2015 WGPS-2, 2018 WGPS-1, and 2018 WGPS-4; KPNP®, Korea: 2015 WGPS-1, 2015 WGPS-3, and 2018 WGPS-2), the weight category (flyweight, featherweight, middleweight, or heavyweight), and the sex of the participant (male or female) were included (Table 1). Furthermore, the ranking position of the athlete during the competition was also taken into consideration.

**Table 1:** Number of matches in each World Grand Prix Series (WGPS) edition.

**Edition**	**Gender**	**Weight category**			
		**Fly**	**Feather**	**Middle**	**Heavy**
		**(*N* = 327)**	**(*N* = 335)**	**(*N* = 341)**	**(*N* = 324)**
Moscow 2015 World Grand Prix Series 1 (*N* = 235)	Men (*N* = 119)	29	30	30	30
	Women (*N* = 116)	29	28	30	29
Samsun 2015 World Grand Prix Series 2 (*N* = 213)	Men (*N* = 109)	24	29	28	28
	Women (*N* = 104)	26	26	25	27
Manchester 2015 World Grand Prix Series 3 (*N* = 207)	Men (*N* = 108)	26	27	27	28
	Women (*N* = 99)	22	25	26	26
Rome 2018 World Grand Prix Series 1 (*N* = 231)	Men (*N* = 113)	28	28	28	29
	Women (*N* = 118)	31	31	31	25
Moscow 2018 World Grand Prix Series 2 (*N* = 211)	Men (*N* = 106)	30	23	30	23
	Women (*N* = 105)	26	30	28	21
Manchester 2018 World Grand Prix Series 4 (*N* = 230)	Men (*N* = 117)	29	30	29	29
	Women (*N* = 113)	27	28	29	29

### Statistical Analysis

Chi-square (χ^2^) tests of association were performed to identify the associations between winners wearing blue and red protectors. The strength of the association was assessed using Pearson's contingency coefficient, the threshold values were classified as weak (0.0–0.3), moderate (0.3–0.6), and strong (>0.6) (Dancey and Reidy, 2007), as well as the odds ratio (OR; Mehta et al., 1985). The effect of wearing blue or red protectors in the whole sample was examined; then, this effect was stratified according to WGPS editions, weight categories, and sex. The potential confounding effect of the first eight seeded athletes (World Taekwondo, 2014b; Falcó et al., 2016), determined by world ranking, and its relation to the wearing of blue or red protectors were analyzed. When a significant relationship was found between being the seeded athletes and winning the match, a binomial logistic regression analysis was performed to analyze the percentage of winning explained. Statistical significance was accepted at *p* < 0.05. The data were tabulated and organized in a Microsoft Excel worksheet and then reported and analyzed using IBM SPSS Statistics for Windows, version 26.0 (IBM Corp., Armonk, NY, USA).

## Results

For the whole sample, the results showed a nonsignificant relationship between the match outcome and the color of the protectors of the winner (χ^2^ = 0.002, *p* = 0.97; *C* = 0.001; OR = 1.00, 95% CI = 0.86–1.17). For individual WGPS (Table 2), the results showed a positive and strong relationship between wearing blue protectors and winning the match in the 2018 WGPS-1 (χ^2^ = 17.53, *p* = 0.001; *C* = 0.19; OR = 2.20, 95% CI = 1.52–3.19) and between wearing red protectors and winning the match in the 2015 WGPS-2 (χ^2^ = 15.78, *p* = 0.001; *C* = 0.19; OR = 0.46, 95% CI = 0.31–0.68).

**Table 2:** Percentage of winners wearing blue and red protectors in each WGPS.

**Edition**	**Color protectors**	
	**Blue (%)**	**Red (%)**
Moscow 2015 World Grand Prix Series 1 (*N* = 235)	51.06	48.94
Samsun 2015 World Grand Prix Series 2 (*N* = 213)	40.38	59.62**
Manchester 2015 World Grand Prix Series 3 (*N* = 207)	45.89	54.11
Rome 2018 World Grand Prix Series 1 (*N* = 231)	59.74**	40.26
Moscow 2018 World Grand Prix Series 2 (*N* = 211)	49.76	50.24
Manchester 2018 World Grand Prix Series 4 (*N* = 230)	52.17	47.83
Overall (*N* = 1,327)	50.04	49.96

Significant association between blue and red color protectors (**p < 0.01).

Regarding the proportion of winners in each WGPS wearing blue or red electronic protectors according to weight categories and sex, the results showed that for men (Table 3), there was a strong relationship between individuals wearing blue and winning the match in the heavyweight category during the 2015 WGPS-2 (χ^2^ = 7.14, *p* = 0.008; *C* = 0.34; OR = 4.46, 95% CI = 1.45–13.68), in the flyweight category during the 2018 WGPS-1 (χ^2^ = 10.29, *p* = 0.001; *C* = 0.39; OR = 6.25, 95% CI = 1.96–19.93), and in the featherweight category during the 2018 WGPS-1 (χ^2^ = 7.14, *p* = 0.008; *C* = 0.34; OR = 4.46, 95% CI = 1.45–13.68). In contrast, the results showed a strong relationship between individuals wearing red and winning the match in the flyweight category during the 2015 WGPS-2 (χ^2^ = 16.33, *p* = 0.001; *C* = 0.50; OR = 0.07, 95% CI = 0.02–0.28) and in the heavyweight category during the 2018 WGPS-2 (χ^2^ = 7.04, *p* = 0.01; *C* = 0.36; OR = 0.19, 95% CI = 0.05–0.67). Then, the results showed a remarkable relationship between individuals wearing blue and winning the match in the middleweight category during the 2015 WGPS-1 (χ^2^ = 4.27, *p* = 0.04; *C* = 0.26; OR = 2.98, 95% CI = 1.04–8.53), in the heavyweight category during the 2018 WGPS-1 (χ^2^ = 5.59, *p* = 0.02; *C* = 0.30; OR = 3.61, 95% CI = 1.22–10.66), and in the flyweight category during the 2018 WGPS-4 (χ^2^ = 5.59, *p* = 0.02; *C* = 0.30; OR = 3.61, 95% CI = 1.22–10.66).

**Table 3:** Percentage of matches won by male athletes according to their color protectors, edition, and weight category.

	**Moscow 2015**	**Samsun 2015**	**Manchester 2015**	**Rome 2018**	**Moscow 2018**	**Manchester 2018**
	**World Grand Prix**	**World Grand Prix**	**World Grand Prix**	**World Grand Prix**	**World Grand Prix**	**World Grand Prix**
	**Series 1**	**Series 2**	**Series 3**	**Series 1**	**Series 2**	**Series 4**
	**(%) (*N* = 119)**	**(%) (*N* = 109)**	**(%) (*N* = 108)**	**(%) (*N* = 113)**	**(%) (*N* = 106)**	**(%) (*N* = 117)**
Fly	Red: 51.72 (29)	Red: 79.17** (24)	Red: 57.63 (26)	Blue: 71.43** (28)	Red: 56.67 (30)	Blue: 65.52* (29)
Feather	Blue: 60 (30)	Red: 62.07 (29)	Blue: 51.85 (27)	Blue: 67.86** (28)	Red: 56.52 (23)	Blue: 60 (30)
Middle	Blue: 63.33* (30)	Blue: 53.57 (28)	Red: 59.26 (27)	Blue: 53.57 (28)	Blue: 56.67 (30)	Blue: 58.62 (29)
Heavy	Red: 53.33 (30)	Blue: 67.86** (28)	Red: 50 (28)	Blue: 65.52* (29)	Red: 69.57** (23)	Red: 62.07 (29)

Significant association between being winner of the match and color protectors (*p < 0.05; **p < 0.01). The number inside brackets means the number of matches in each weight category.

Similarly, for women (Table 4), the results showed a strong and significant relationship between individuals wearing blue and winning the match in the featherweight category during the 2018 WGPS-1 (χ^2^ = 23.29, *p* = 0.001; *C* = 0.52; OR = 17.36, 95% CI = 4.92–61.21) and in the flyweight category during the 2018 WGPS-2 (χ^2^ = 11.08, *p* = 0.001; *C* = 0.42; OR = 7.37, 95% CI = 2.16–25.09). On the contrary, a significant and strong relationship was found between individuals wearing red protectors and winning the match in the flyweight category during the 2015 WGPS-2 (χ^2^ = 11.08, *p* = 0.001; *C* = 0.42; OR = 0.14, 95% CI = 0.04–0.46) and in the middleweight category during the 2015 WGPS-2 (χ^2^ = 18.00, *p* = 0.001; *C* = 0.51; OR = 0.06, 95% CI = 0.02–0.25). Moreover, a moderate and significant relationship was found between individuals wearing red and winning the match in the featherweight category during the 2015 WGPS-1 (χ^2^ = 4.57, *p* = 0.03; *C* = 0.27; OR = 0.31, 95% CI = 0.10–0.92).

**Table 4:** Percentage of matches won by female athletes according to their color protectors, edition, and weight category.

	**Moscow 2015**	**Samsun 2015**	**Manchester 2015**	**Rome 2018**	**Moscow 2018**	**Manchester 2018**
	**World Grand Prix**	**World Grand Prix**	**World Grand Prix**	**World Grand Prix**	**World Grand Prix**	**World Grand Prix**
	**Series 1**	**Series 2**	**Series 3**	**Series 1**	**Series 2**	**Series 4**
	**(%) (*N* = 116)**	**(%) (*N* = 104)**	**(%) (*N* = 99)**	**(%) (*N* = 118)**	**(%) (*N* = 105)**	**(%) (*N* = 113)**
Fly	Red: 51.72 (29)	Red: 73.08** (26)	Blue: 54.55 (22)	Blue: 51.61 (31)	Blue: 73.08** (26)	Blue: 55.56 (27)
Feather	Red: 64.29* (28)	Red: 57.69 (26)	Red: 55.56 (25)	Blue: 80.65** (31)	Red: 53.33 (30)	Blue: 56.14 (28)
Middle	Blue: 53.33 (30)	Red: 80** (25)	Red: 61.54 (26)	Red: 58.06 (31)	Red: 53.57 (28)	Red: 58.62 (29)
Heavy	Blue: 51.72 (29)	Red: 51.85 (27)	Red: 53.85 (26)	Red: 56 (25)	Blue: 57.14 (21)	Red: 58.62 (29)

Significant association between being winner of the match and color protectors (*p < 0.05; **p < 0.01). The number inside brackets means the number of matches in each weight category.

Additionally, the potential confounding effect of being a seeded athlete was analyzed in those weight categories in which the color effect was significant [for the figures with nonsignificant relationships between being a seeded athlete and winning the match (Supplementary Material)].

In the male heavyweight category during the 2015 WGPS-2 (Supplementary Figure 3), a nonsignificant relationship was found between being a seeded athlete and winning the match (*C* = 0.03; OR = 0.88, 95% CI = 0.32–2.38) nor was a significant relationship found between being a seeded athlete and the color of the protectors (*C* = 0.20; OR = 2.25, 95% CI = 0.52–9.70).

In the male flyweight category during the 2018 WGPS-1 (Supplementary Figure 4), a nonsignificant relationship was found between being a seeded athlete and winning the match (*C* = 0.10; OR = 0.68, 95% CI = 0.25–1.84). However, a significant relationship was found between being a seeded athlete and the color of the protectors being blue (*C* = 0.39; OR = 6.25, 95% CI = 1.21–32.22).

In the male featherweight category during the 2018 WGPS-1 (Supplementary Figure 5), a nonsignificant relationship was found between being a seeded athlete and winning the match (*C* = 0.16; OR = 1.92, 95% CI = 0.70–5.26), whereas a significant and strong relationship was found between being a seeded athlete and the color of the protectors being blue (*C* = 0.41; OR = 6.76, 95% CI = 1.57–29.07).

**Figure 1:**
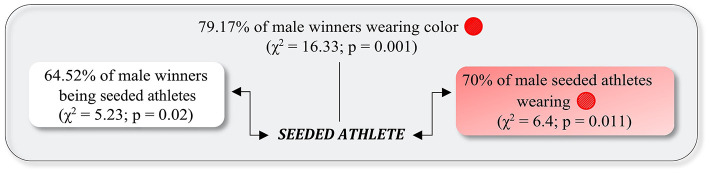
Relationship between the color of the protectors and the result of the match, considering seeded athletes as a moderation factor, for male athletes in flyweight, Samsun 2015 World Grand Prix Series 2. Continuous lines indicate significant relationships.

In the male flyweight category during the 2015 WGPS-2 (Figure 1), a strong and significant relationship was found between being a seeded athlete and winning the match (*C* = 0.28; OR = 3.31, 95% CI = 1.17–9.36), and similarly, a strong and significant relationship was found between being a seeded athlete and the color of the protectors being red (*C* = 0.37; OR = 0.18, 95% CI = 0.05–0.71). The logistic regression model was statistically significant, $\chi_{\left(2\right)}^{2}$ = 24.23, *p* < 0.001. The model, containing the seeded athlete and the color of the protectors, explained 48% (Nagelkerke *R*^2^) of the variance in winning the match and correctly classified 74.5% of cases.

In the male heavyweight category during the 2018 WGPS-2 (Supplementary Figure 6), a nonsignificant relationship was found between being a seeded athlete and winning the match (*C* = 0.10; OR = 1.47, 95% CI = 0.54–4.01) nor was a significant relationship found between being a seeded athlete and the color of the protectors (*C* = 0.28; OR = 0.30, 95% CI = 0.07–1.21).

In the male middleweight category during the 2015 WGPS-1 (Supplementary Figure 7), a strong and negative relationship was found between being a seeded athlete and winning the match (*C* = 0.33; OR = 0.23, 95% CI = 0.08–0.66), and a significant and strong relationship was found between being a seeded athlete and the color of the protectors being blue (*C* = 0.51; OR = 16.00, 95% CI = 1.79–143.16).

**Figure 2:**
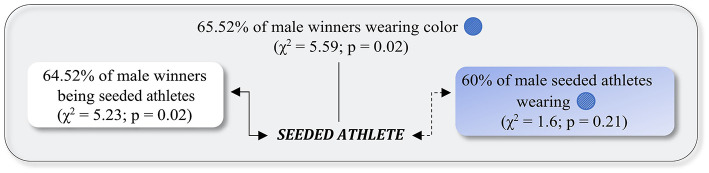
Relationship between the color of the protectors and the result of the match, considering seeded athletes as a moderation factor, for male athletes in heavyweight, Rome 2018 World Grand Prix Series 1. Continuous lines indicate significant relationships. Discontinuous lines indicate non-significant relationships.

In the male heavyweight category during the 2018 WGPS-1 (Figure 2), a strong and significant relationship was found between being a seeded athlete and winning the match (*C* = 0.28; OR = 3.31, 95% CI = 1.17–9.36), but no significant relationship was found between being a seeded athlete and the color of the protectors (*C* = 0.20; OR = 2.25, 95% CI = 0.63–7.97). The logistic regression model was statistically significant, $\chi_{\left(2\right)}^{2}$ = 17.21, *p* < 0.001. The model, containing the seeded athlete and the color of the protectors, explained 33% (Nagelkerke *R*^2^) of the variance in winning the match and correctly classified 71.7% of cases.

In the male flyweight category during the 2018 WGPS-4 (Supplementary Figure 8), a nonsignificant relationship was found between being a seeded athlete and winning the match (*C* = 0.03; OR = 0.88, 95% CI = 0.32–2.38) nor was a significant relationship found between being a seeded athlete and the color of the protectors (*C* = 0.32; OR = 4.00, 95% CI = 0.88–18.26).

In the female featherweight and category during the 2018 WGPS-1 (Supplementary Figure 9), a nonsignificant relationship was found between being a seeded athlete and winning the match (*C* = 0.16; OR = 1.92, 95% CI = 0.70–5.26), whereas a significant and strong relationship was found between being a seeded athlete and the color of the protectors being blue (*C* = 0.49; OR = 12.25, 95% CI = 2.54–58.97).

In the female flyweight category during the 2018 WGPS-2 (Supplementary Figure 10), a nonsignificant relationship was found between being a seeded athlete and winning the match (*C* = 0.03; OR = 0.88, 95% CI = 0.32–2.38), but a significant and strong relationship was found between being a seeded athlete and the color of the protectors being blue (*C* = 0.42; OR = 7.56, 95% CI = 1.50–38.15).

In the female flyweight category during the 2015 WGPS-2 (Supplementary Figure 11), a nonsignificant relationship was found between being a seeded athlete and winning the match (*C* = 0.03; OR = 1.14, 95% CI = 0.42–3.08), and similarly, a nonsignificant relationship was found between being a seeded athlete and the color of the protectors (*C* = 0.24; OR = 0.36, 95% CI = 0.09–1.51).

In the female middleweight category during the 2015 WGPS-2 (Supplementary Figure 12), a nonsignificant relationship was found between being a seeded athlete and winning the match (*C* = 0.22; OR = 2.51, 95% CI = 0.90–6.97), whereas a significant and strong relationship was found between being a seeded athlete and the color of the protectors being red (*C* = 0.50; OR = 0.07, 95% CI = 0.01–0.34).

In the female featherweight category during the 2015 WGPS-1 (Supplementary Figure 13), a nonsignificant relationship was found between being a seeded athlete and winning the match (*C* = 0.10; OR = 0.68, 95% CI = 0.25–1.84) nor was a significant relationship found between being a seeded athlete and the color of the protectors (*C* = 0.14; OR = 0.56, 95% CI = 0.13–2.51).

## Discussion

The objective of this study was to analyze the relationship between color protectors and success in 1,327 taekwondo matches from 6 WGPS of two 4-year Olympic periods. This is the first time that the influence of color has been analyzed using electronic protectors for both the body and the head. Analyses were performed for the total sample, which was also examined according to editions, weight categories, and sex. In addition, we analyzed the confounding effect of being a seeded athlete in these competitions.

When analyzing the total sample, the results did not show a relationship between the match outcome (winning or losing the match) and the color of the electronic protectors of the winner. Our results are in line with the studies by Carazo-Vargas and Moncada-Jiménez (2014) and Falcó et al. (2016) who analyzed the 2013 World Championship and 2012 Olympic Games (including the Qualification Tournaments using the same system), respectively. Carazo-Vargas and Moncada-Jiménez (2014) studied 812 matches (male = 490 and female = 322) with the one-sample binomial test that revealed similar win rates when wearing blue or red protectors (*p* = 0.48), while Falcó et al. (2016) analyzed 462 matches (male = 245 and female = 217), and their results showed a non-significant relationship between the match outcome and the color of the protectors of the winner (*p* = 0.14). On the contrary, our results are in contrast to the findings made by Hill and Barton (2005), analyzing the 2004 Olympic Games, which found that 43 of 75 matches were won by athletes wearing red protectors (*p* < 0.05). This can be due to the scoring system used and the system to assign red or blue protectors. That is, Hill and Barton (2005) analyzed a competition that used a manual scoring system, and blue and red protectors were assigned randomly. Carazo-Vargas and Moncada-Jiménez (2014) and Falcó et al. (2016) analyzed competitions that used an electronic body (but not helm) scoring system, and the color of the protectors were assigned based on a ranked system. Carazo-Vargas and Moncada-Jiménez (2014) suggested that previous research might have been biased by not taking into consideration the human error in refereeing, a phenomenon described before (Hagemann et al., 2008; Lopez and Snyder, 2013) and that the electronic scoring systems seem to solve.

For the six WGPS, the results showed a positive and strong relationship in two of them: one between wearing blue protectors and winning a match in the 2018 WGPS-1 and the other between wearing red protectors and winning a match in the 2015 WGPS-2. Our results are in contrast to the study by Feltmann and Elliot (2011) who found that participants, who are imagining themselves wearing red in a taekwondo match, had enhanced self-perception of their own dominance and threat, whereas perceiving an opponent in red enhanced the perception of their dominance and threat. Falcó et al. (2016) also found a positive, but weak, relationship between wearing red protectors and winning matches in the Asian (58.6%, *N* = 99) and European (56.7%, *N* = 120) Qualification Tournaments but neither in the Pan American (46.2%, *N* = 91) nor in the 2012 Olympic Games (47.4%, *N* = 152). The authors tried to explain the advantage of wearing red in these two tournaments through a combination of two factors: First, related to the people (i.e., the referees) who judged the performance (Hagemann et al., 2008; Sorokowski et al., 2014), and second, related to the methods of interpreting rules and regulations and the application of judgment criteria (Myers et al., 2010; Sorokowski et al., 2014). They concluded that even though the electronic body protectors awarded the points for techniques performed on the trunk, a subjective judgment by the referees was still present by highlighting the need to continue with the implementation of a fully objective system that includes the electronic registration of points for all the areas in which it is permitted to score in taekwondo. In line with the conclusions of the studies by Carazo-Vargas and Moncada-Jiménez (2014) and Falcó et al. (2016), our study suggests that the further introduction of the electronic helmet has made the scoring system more objective with very similar win rates (between blue and red) when analyzing the total sample and a more balanced situation (between blue and red) when analyzing individual WGPS editions.

Regarding the proportion of winners wearing blue or red electronic protectors according to weight categories, 8 and 5 of 48 showed significantly higher percentages of blue and red winners, respectively. Moreover, the proportion of significant blue winners were 2 and 6 of 48 in the 2015 and 2018 WGPS editions, while the proportion of significant red winners were 4 and 1 of 48 in the 2015 and 2018 WGPS editions, respectively. In the 2004 Athens Olympic Games, Hill and Barton (2005) only found a significant relationship in some weight categories between red color and winning, with no classes having significantly more blue winners. Falcó et al. (2016) found that, from 32 weight categories, the matches in 3 categories were systematically won by players wearing blue, and similarly, the matches in 6 categories were systematically won by players wearing red. Placing our results in perspective, it can be observed a slight tendency to an overhanging of color effect from red to blue. The results extend the evidence (Hagemann et al., 2008) that when referees do not give the points to the athletes, it seems that wearing blue (and seeing red) might also benefit the performance. With the inclusion of the electronic helmet, the points given by the referees are reduced to punches and penalties. Therefore, it is possible that when the influence of the referees in judging the performance is limited (i.e., an electronic score system is instead used), and following the color-in-context theory (Elliot and Maier, 2014), seeing red has a stronger influence to increase the performance than wearing red. Therefore, the electronic scoring system seems to be a good tool to decrease the advantage of referees toward the red wearer in winning matches.

Regarding sex, male competitors showed a positive significant relationship between blue color and systematically winning the match in 6 of 24 weight categories. On the contrary, the relationship between wearing red and winning the match was significantly shown in 2 of 24 weight categories. In the 2004 Athens Olympic Games, the proportion of weight categories won by male athletes wearing red was 4 of 4, with no weight classes having significantly more blue winners (Hill and Barton, 2005). In 2012, the proportion of weight categories systematically won by male contenders wearing blue was 2 of 16, while the proportion wearing red was significantly won by 1 of the 16 weight categories (Falcó et al., 2016). Female competitors showed a positive significant relationship between blue color and winning the match in 2 of 24 weight categories while wearing red and winning the match was systematically shown in 3 of 24 weight categories. Hill and Barton (2005) did not provide data regarding female contenders, while Vasconcelos and Del Vecchio (2017) found a strong red color effect (65% of the matches) in female Wushu Sanda athletes, with a manual scoring system. Falcó et al. (2016) found that, in 1 and 5 of 16 weight categories, the matches were systematically won by female athletes wearing blue and red, respectively. But Goldschmied and Spitznagel (2021) did not find a relationship between uniform colors and success in the elite Women's NCAA Basketball Tournaments. From the results of this study, it seems that men are slightly more sensitive or seem to be more affected by the color of the protector (by switching from red to blue) than women, which highlights the importance of gender in analyzing the color effect.

When it comes to the influence of being a seeded athlete on winning the match, the results of this study did show a significant confounding effect on the color of the protectors worn by the winner of the match in 2 of 13 weight categories. In the male flyweight category during the 2015 WGPS-2 and the male heavyweight category during the 2018 WGPS-1, the model explained 48 and 33% of the variance in winning the match and correctly classified 74.5 and 71.7% of cases, respectively. These results confirm and extend what was found by Falcó et al. (2016) in the 2012 Olympic Games, where a significant confounding effect on the color of the protectors worn by the winner of the match emerged in 1 of 3 weight categories. The difficulty in discriminating between seeded and nonseeded athletes in the WGPS and the decrease of this effect compared to the 2012 Olympic Games could be due to the fact that: (1) the WT introduced seeding based on world rankings shortly before the London 2012 Olympics (World Taekwondo, 2010) and (2) the WT introduced the qualification system for the Olympic Games through world ranking after the London 2012 Olympics (World Taekwondo, 2015). In this context, the WGPS is a closed-numbered event introduced in 2013, in which the top 31 athletes of the world ranking and 1 athlete from the host country of the edition per weight category take part (World Taekwondo, 2014b). Based on the abovementioned context and our results, it emerges that the reduced influence of being a seeded athlete on winning the match equalizes the athletes who participate in the WGPS, confirming the very purpose of the competition.

## Limitations and Future Research Lines

Although this study considered a larger number of matches and involved a time frame of two 4-year Olympic periods, compared to past studies conducted on a smaller number of matches and individual competitions (Hill and Barton, 2005; Carazo-Vargas and Moncada-Jiménez, 2014; Falcó et al., 2016), some limitations should be considered. First, our results cannot be generalized to the population as we have analyzed only a closed-numbered event, such as the WGPS (World Taekwondo, 2014b), in which the top athletes of the world ranking with similar competitive levels take part. To this end, future studies are encouraged to also analyze competitions with a different participation system in which not only top-ranked athletes but also athletes of different competitive levels (e.g., Olympic Games, World Championships, and Continental Championships) take part. Second, the WGPS, being a competition introduced in 2013 (World Taekwondo, 2014b), has since the first edition used at least one electronic device (i.e., body protector) for the scoring system, making it impossible for this competition to compare manual and electronic scoring systems. Therefore, future research is recommended to consider analyzing one or more competitions (e.g., Olympic Games, World Championships, and Continental Championships) over a time frame long enough to include the use of both the manual and electronic scoring systems, thus allowing for a direct comparison between the two systems. This could help identify and quantify the possible presence of human error related to refereeing as previously hypothesized by Carazo-Vargas and Moncada-Jiménez (2014) and Falcó et al. (2016). Finally, the difficulty in identifying the influence of the confounding factor for winning the match in the WGPS, given its characteristics, brings out the need to use an alternative method for this purpose. Hill and Barton (2005) used the analysis of varying degrees of asymmetry to quantify the role of confounding factors, such as skill and strength, among athletes. They found that there were significantly more red than blue winners only in matches between individuals of similar ability, with the red advantage seeming to decline as asymmetries in competitive ability increase. To this end, the method of analyzing varying degrees of asymmetry proposed by Hill and Barton (2005) could be an alternative solution for quantifying the confounding factor in world-class competitions, as this method relies on coding matches into different classes of asymmetry based on the difference in points scored by each athlete in a given competition; in contrast, the method used in our study and by Falcó et al. (2016) is based on the world ranking points acquired in the previous 4-year Olympic period and not on the degree of skill and strength of an athlete on the day of a given competition.

## Conclusion

Our study suggests that the objectification of the scoring system, with the introduction of electronic protections for all areas where scoring is allowed in the taekwondo matches, allows for more fair or equal competition. Therefore, the electronic scoring system seems to be a good tool to decrease the advantage of referees toward the red wearer in winning matches. Regarding weight categories, it seems that some of them are more sensitive to note the color while others are more affected by wearing the color. Also, regarding sex, it seems that male contenders are more likely to be influenced by the color protector than female contenders. Finally, being a seeded athlete did not emerge as a predictive factor for winning the match in WGPS; the difficulty in discriminating between seeded and nonseeded athletes equalizes the athletes who participate in the WGPS, confirming the very purpose of the competition.

## Data Availability Statement

Publicly available datasets were analyzed in this study. This data can be found at: http://m.worldtaekwondo.org/competition/list.html?mcd=A01&sc=re.

## Author Contributions

GA and CF conceived and designed the study. GA collected the data and analyzed them. CF contributed to the interpretation of the results and also reviewed and provided feedback to the manuscript. Both authors contributed substantially to the final version of the manuscript and approved it for publication.

## Funding

This publication was financed by the Western Norway University of Applied Sciences.

## Conflict of Interest

The authors declare that the research was conducted in the absence of any commercial or financial relationships that could be construed as a potential conflict of interest.

## Publisher's Note

All claims expressed in this article are solely those of the authors and do not necessarily represent those of their affiliated organizations, or those of the publisher, the editors and the reviewers. Any product that may be evaluated in this article, or claim that may be made by its manufacturer, is not guaranteed or endorsed by the publisher.
