# Supplementary material for: When Taekwondo Referees See Red, but It Is an Electronic System That Gives the Points
Source: Front Psychol. 2021 Dec 13;12:787000. doi: 10.3389/fpsyg.2021.787000 (PMC8710472; doi:10.3389/fpsyg.2021.787000)
Supplement: Supplementary file 1 [file Data_Sheet_1.PDF]

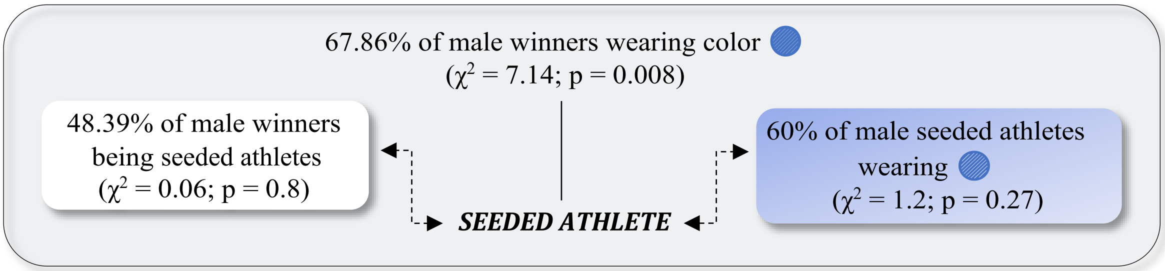

Supplementary Figure 3. Relationship between the color of the protectors and the result of the match, considering seeded athletes as a moderation factor, for male athletes in heavyweight, Samsun 2015 World Grand Prix Series 2. Discontinuous lines indicate non-significant relationships.

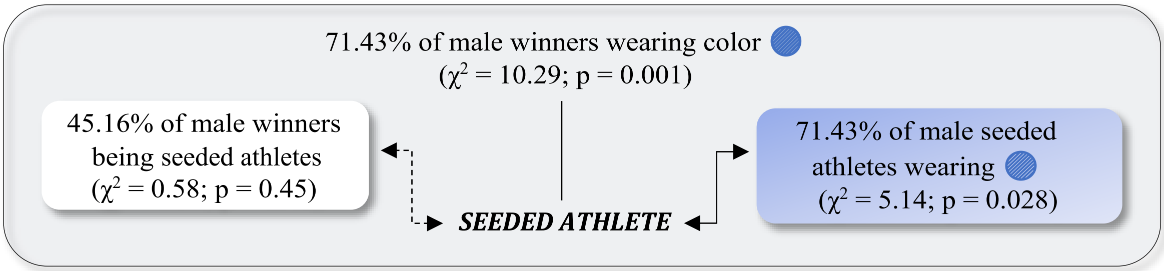

Supplementary Figure 4. Relationship between the color of the protectors and the result of the match, considering seeded athletes as a moderation factor, for male athletes in flyweight, Rome 2018 World Grand Prix Series 1. Continuous lines indicate significant relationships. Discontinuous lines indicate non-significant relationships.

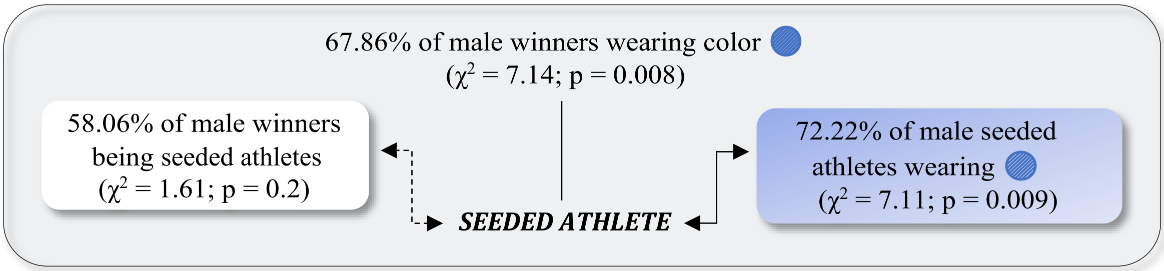

Supplementary Figure 5. Relationship between the color of the protectors and the result of the match, considering seeded athletes as a moderation factor, for male athletes in featherweight, Rome 2018 World Grand Prix Series 1. Continuous lines indicate significant relationships. Discontinuous lines indicate non-significant relationships.

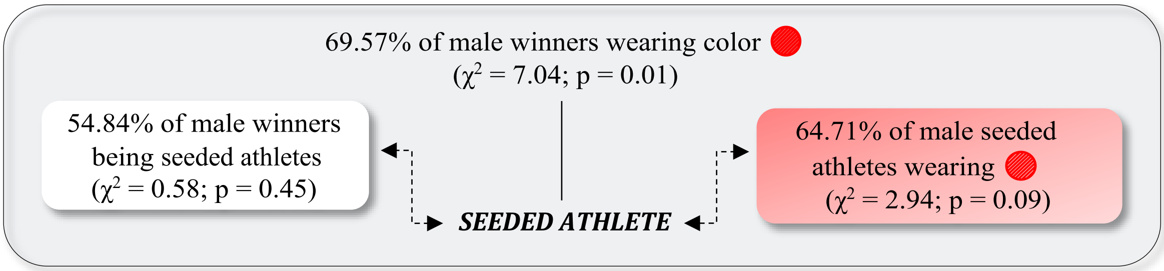

Supplementary Figure 6. Relationship between the color of the protectors and the result of the match, considering seeded athletes as a moderation factor, for male athletes in heavyweight, Moscow 2018 World Grand Prix Series 2. Discontinuous lines indicate non-significant relationships.

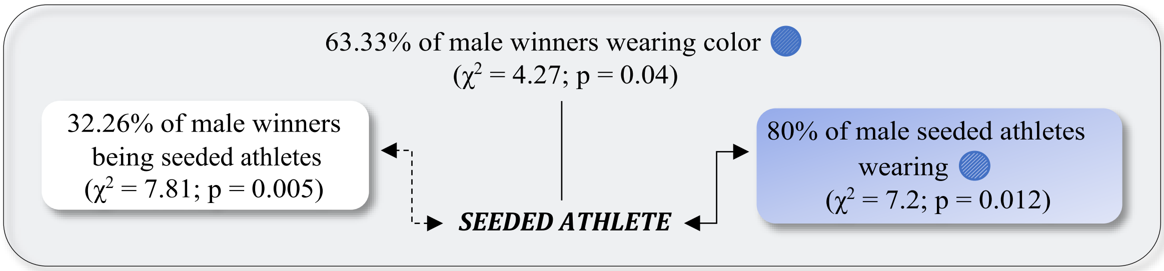

Supplementary Figure 7. Relationship between the color of the protectors and the result of the match, considering seeded athletes as a moderation factor, for male athletes in middleweight, Moscow 2015 World Grand Prix Series 1. Continuous lines indicate significant relationships.

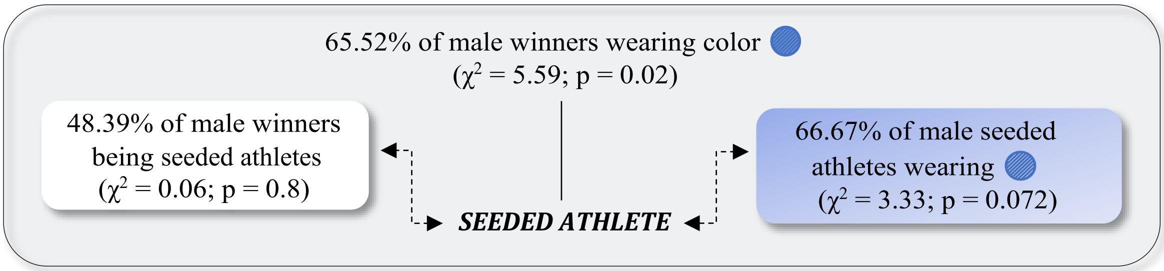

Supplementary Figure 8. Relationship between the color of the protectors and the result of the match, considering seeded athletes as a moderation factor, for male athletes in flyweight, Manchester 2018 World Grand Prix Series 4. Discontinuous lines indicate non-significant relationships.

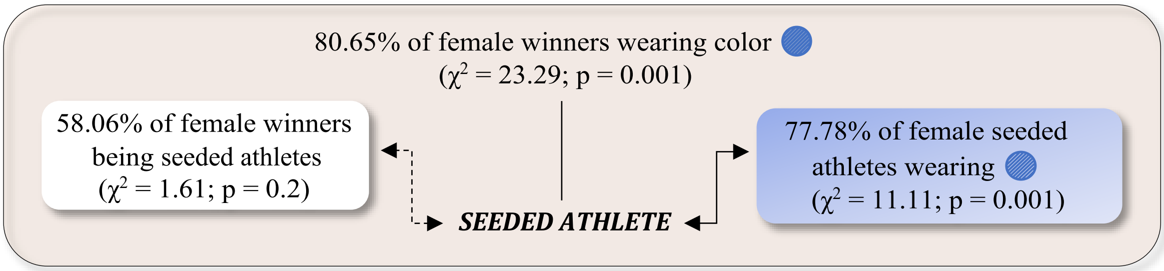

Supplementary Figure 9. Relationship between the color of the protectors and the result of the match, considering seeded athletes as a moderation factor, for female athletes in featherweight, Rome 2018 World Grand Prix Series 1. Continuous lines indicate significant relationships. Discontinuous lines indicate non-significant relationships.

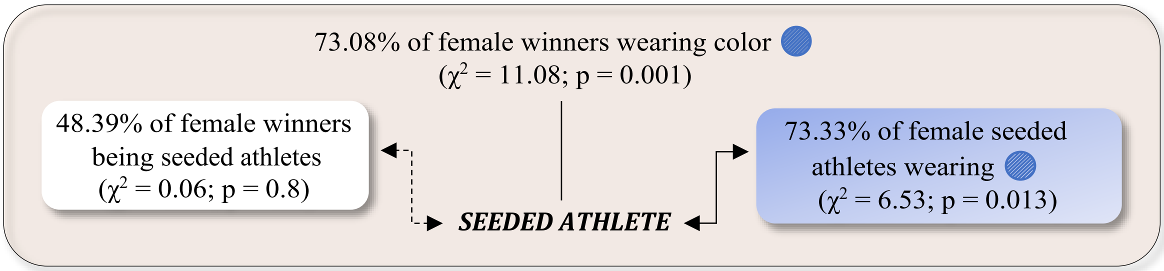

Supplementary Figure 10. Relationship between the color of the protectors and the result of the match, considering seeded athletes as a moderation factor, for female athletes in flyweight, Moscow 2018 World Grand Prix Series 2. Continuous lines indicate significant relationships. Discontinuous lines indicate non-significant relationships.

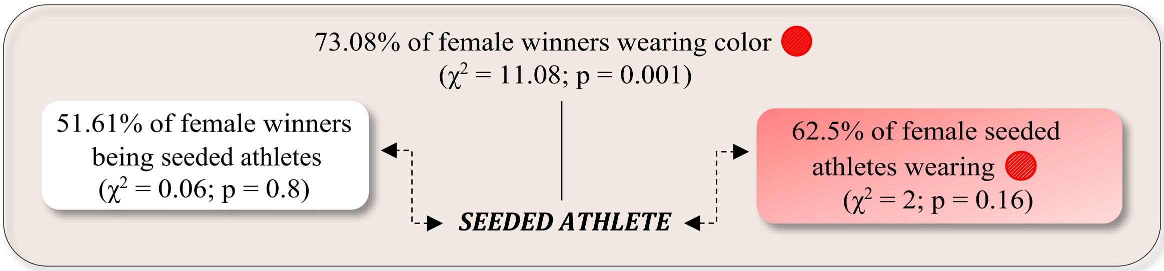

Supplementary Figure 11. Relationship between the color of the protectors and the result of the match, considering seeded athletes as a moderation factor, for female athletes in flyweight, Samsun 2015 World Grand Prix Series 2. Discontinuous lines indicate non-significant relationships.

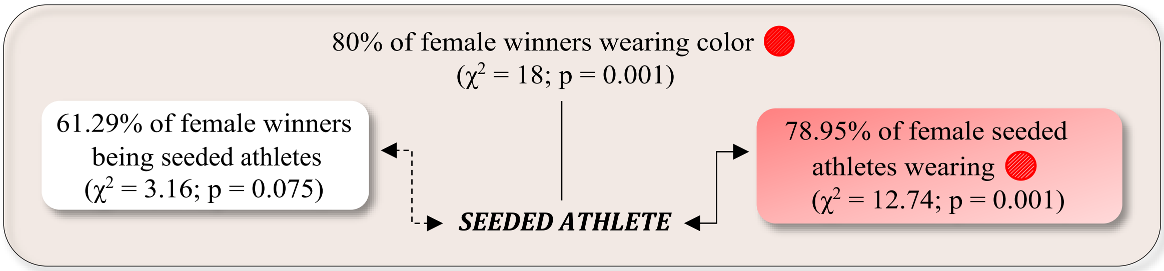

Supplementary Figure 12. Relationship between the color of the protectors and the result of the match, considering seeded athletes as a moderation factor, for female athletes in middleweight, Samsun 2015 World Grand Prix Series 2. Continuous lines indicate significant relationships. Discontinuous lines indicate non-significant relationships.

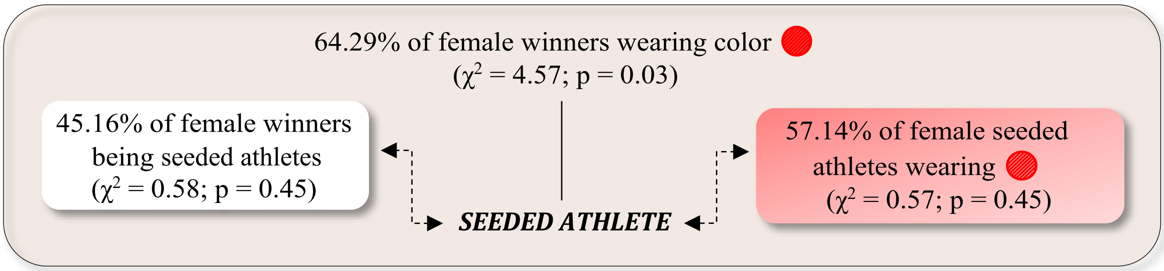

Supplementary Figure 13. Relationship between the color of the protectors and the result of the match, considering seeded athletes as a moderation factor, for female athletes in featherweight, Moscow 2015 World Grand Prix Series 1. Discontinuous lines indicate non-significant relationships.
